# Co-Morbidity Clusters in Post-COVID-19 Syndrome

**DOI:** 10.3390/jcm13051457

**Published:** 2024-03-02

**Authors:** Anna Teréz Sárközi, Ilona Tornyi, Erik Békési, Ildikó Horváth

**Affiliations:** 1 Department of Pulmonology, University of Debrecen, 4032 Debrecen, Hungary; sarkozi.anna@med.unideb.hu (A.T.S.); tornyi.ilona@med.unideb.hu (I.T.); bekesierik97@gmail.com (E.B.); 2 National Koranyi Institute of Pulmonology, 1121 Budapest, Hungary

**Keywords:** SARS-CoV-2, post-acute COVID-19 syndrome, long COVID, multidisciplinary team, multiorgan syndrome

## Abstract

**Background:** Post-COVID-19 syndrome, characterized by persistent symptoms emerging more than 12 weeks after acute infection, displays diverse manifestations. This study aimed to analyze co-existing organ dysfunctions in post-COVID-19 patients and explore their potential association with the acute COVID-19 episode and functional impairment. **Methods:** Data from 238 patients attending post-COVID-19 outpatient care between 1 March 2021 and 1 March 2022, after previous hospitalization for acute COVID-19, were retrospectively analyzed with 80 having comprehensive mapping of organ involvement. **Results:** The average time between acute episode and post-COVID-19 care was 149 days. Spirometry indicated significant abnormalities in lung function. Predominant symptoms included respiratory (75%), fatigue (73%), neurological (62.5%), and ear-nose-throat issues (51.25%). Multiorgan dysfunctions were observed in 87.5% of patients, contributing to an 18.33% reduction in health quality compared to pre-acute COVID-19 levels. Subgroup analysis identified four distinct post-COVID-19 syndrome subgroups, highlighting the coexistence of respiratory and neurological disorders as potential indicators and drivers of further organ involvement. Our results reveal that most patients with post-COVID-19 syndrome suffer from multiorgan disorders. **Conclusions:** The presence of coexisting respiratory and neurological symptoms suggests the involvement of other organ systems as well. The complexity of multiorgan involvement requires further studies to provide insights into the different symptom clusters and identify potential targets for personalized preventive and therapeutic interventions to improve patient outcome.

## 1. Introduction

As the world continues to struggle with the aftermath of the COVID-19 pandemic, a new phenomenon has emerged: post-COVID-19 syndrome. This condition, also known as long COVID, affects individuals who have recovered from the acute phase of infection but continue to experience a wide array of symptoms persisting or appearing after the initial 12 weeks following the onset of acute COVID-19 [1]. New challenges occur in dealing with post-COVID-19 syndrome; millions of people require medical care because 10–45% of those who have had the infection develop post-COVID-19 syndrome [2,3,4].

Post-COVID-19 syndrome presents a wide range of symptoms, including general signs (fatigue and widespread pain), respiratory symptoms (shortness of breath), psychiatric disorders (post-traumatic stress syndrome, depression, anxiety, and insomnia), as well as neurological problems (cognitive impairment) [4,5,6,7]. COVID-19 is a respiratory tract infection that has diverse pulmonary manifestations from mild symptoms (cough or sore throat) to moderate disease (featuring pneumonia or different signs of acute lung injury) to severe, life-threatening, or fatal acute respiratory distress syndrome (ARDS) [8]. Moreover, specific organ damage can also affect systems like cardiovascular, renal, endocrine, ear-nose-throat, gastrointestinal, and dermatological systems. Individuals with post-COVID-19 syndrome commonly experience multiple overlapping symptoms, significantly diminishing their quality of life [9,10]. The exact course and mechanisms of these lingering symptoms remain elusive, posing a significant challenge for both medical professionals and researchers [11].

Due to the diverse range of symptoms and their fluctuating nature, diagnosis often proves elusive, leaving many patients frustrated and underserved [12,13]. Additionally, the absence of specific treatments exacerbates the situation, leaving healthcare providers with limited options for managing the condition effectively. Increased demand for specialized care, diagnostics, and rehabilitation services strain resources. Moreover, the long-term nature of the condition emphasizes the importance of holistic and multidisciplinary approaches to address the complex needs of patients [14].

**Figure 1:**
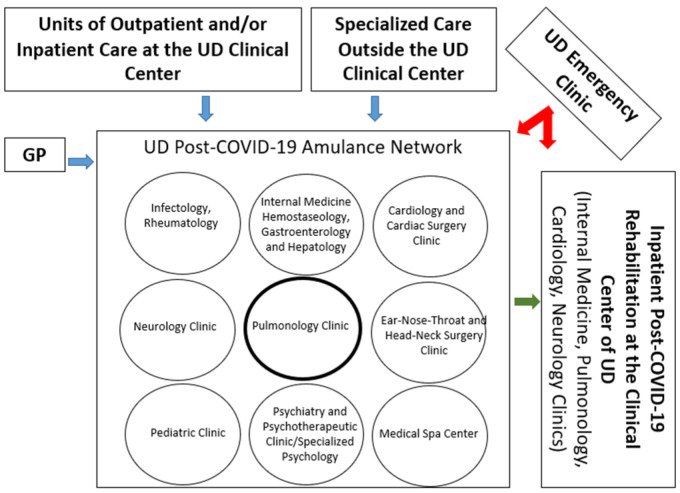
Organizational chart of the Post-COVID Specialized Outpatient Clinics at the UD Clinical Centre.

At the beginning of 2021 when the world started to identify the long-term consequences of the first and the second wave of the pandemic, there was no uniform guideline for post-COVID-19 care. In order to provide specialized care for these patients, the Clinical Centre of the University of Debrecen (UD) established a Post-COVID Outpatient Service (PCOS) starting from the 1 March 2021. Based on the frequency of post-COVID-19 clinical symptoms and complaints, specialized areas were invited to provide experts and form a multidisciplinary team for post-COVID-19 care. UD established its PCOS including pulmonology, neurology, psychiatry, cardiology, infectology, hematology, internal medicine, rheumatology, and rehabilitation (Figure 1). It was advised that GPs refer the patient based on the dominant leading complaint/symptom through a given specialty to the service. Then, the first attending PCOS member managed the patient’s pathway according to his/her need.

As we know little about the exact nature of post-COVID-19 syndrome up to now, we assessed the diversity of complaints presented by patients at the UD Pulmonology Clinic, a part of the UD PCOS, over its first year. The primary aim of this study was to analyze the characteristics of symptoms and the patterns of co-occurrence of organ dysfunctions to attempt to subgroup this syndrome based on real-world data from our outpatient clinic.

## 2. Materials and Methods

### 2.1. Study Population and Design

**Figure 2:**
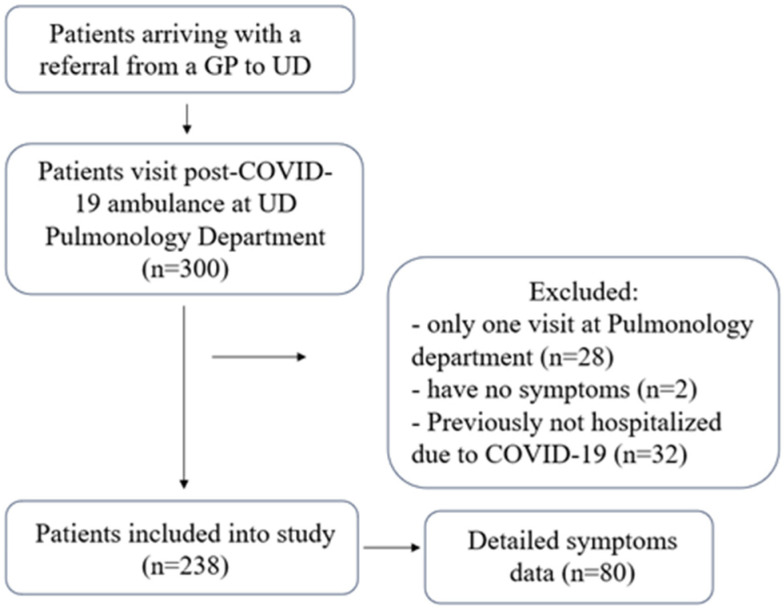
Participant inclusion process.

Data on patients who had been hospitalized during their acute COVID-19 episode, had remaining unresolved symptoms or developed new symptoms unexplained by other diseases afterwards, and attended our clinic at least twice, were collected and analyzed in this study. Subjects were referred by their GPs with various symptoms after their acute COVID-19 episode to the UD Pulmonology Outpatient Clinic, Debrecen, Hungary as part of the UD PCOS network between 1 March 2021 and 1 March 2022 (Figure 2). 

The present study was conducted in accordance with the principles of the Declaration of Helsinki and after the approval by the regional and the local institutional Ethical Committee of the University of Debrecen (DEOEC RKEB/IKEB 5959-2022).

Demographic characteristics (age, sex) and clinical features (comorbidities, post-COVID-19 symptoms) were retrieved from institutional electronic medical records. The definition of post-COVID-19 used at our clinic was identical to how Nalbandian et al. defined it [1]. 

### 2.2. Lung Function 

Lung function tests were performed in a whole-body plethysmography box (PDT-111/pd, Piston Medical, Budapest, Hungary). Algorithms provided by the manufacturer were used to record raw pulmonary function data and calculate the percentage of normal reference values. The following parameters were collected: diffusion capacity (DLCO), forced expiratory volume in one second (FEV1), forced vital capacity (FVC), and Tiffeneau-index (FEV1/FVC). 

### 2.3. CAT Score Measurement

CAT is a short questionnaire that was filled out by patients to measure the health status of patients with chronic obstructive pulmonary diseases (COPD) [15,16]. CAT score reflects cough, sputum, chest tightness, breathlessness due to physical activity (walking up on stairs), everyday activities at home, confidence to leave home, sleep quality, and energy levels in an aggregated way. Scores may vary between 0 and 40. While low scores mean no or mild health effect of the lung disease, higher scores mean a worse health state due to the symptoms related to lung functional impairment. As a validated test with a good discriminative value in COPD, and no such validated test developed for post-COVID-19, we used it in our post-COVID-19 care to assess the health state in relation to pulmonary function [17]. The official Hungarian translation of the CAT was filled out by the patients at their post-COVID-19 visit [15]. The questionnaire is publicly available in both Hungarian [16] and English [18] languages.

### 2.4. Measurement of Quality of Life 

Quality of life was assessed using the Visual Analog Scale (VAS) [19,20]. On this scale, the patient’s quality of life before and after SARS-CoV-2 infection was subjectively rated on a scale ranging from 1 to 100. The VAS questionnaire was filled out when patients appeared at the PCOS. They were requested to score their life quality before the acute episode of COVID-19 and after COVID-19 as felt at the time of their PCOS visit.

### 2.5. Statistics

The Shapiro–Wilk test was used to assess the normality of continuous variables. When the data followed a Gaussian distribution, a Student’s t-test was employed for comparing two datasets, and the results were presented as mean ± SD. If the distribution was not Gaussian, the Mann–Whitney U-test was used, and the data were presented as median with interquartile range (IQR). The result was considered significant when the p-value was less than 0.05.

## 3. Results

Between 1 March 2021 and 1 March 2022, a total of 238 individuals presented with post-COVID-19 symptoms at the respiratory unit of the PCOS network (Table 1). 

The mean age of patients was 55 years and there was a slight predominance of females. Patients were referred by GPs and were presented at the PCOS an average of 149 days after recovering from the acute episode of COVID-19. All patients were hospitalized for their previous acute COVID-19 episode. Most of them had one or more preexisting medical conditions (Table 1). Almost half of these patients had coexisting cardiovascular disease, and one fifth of them had some kind of chronic respiratory disorder.

### 3.1. Lung Function Tests

FEV1 data measured at the post-COVID-19 visit were available for 191 individuals. Out of these, 38 (19.90%) had lower than normal values (<80% predicted), while 153 (80.10%) had normal values. The Tiffeneau index was <80% in 58.82% of them. DLCO values were measured for 59 patients. Among them, 23 (38.98%) had low values (<75% predicted; Table 2).

Spirometric data were compared with age, gender, and days of hospitalization. There was a significant relationship between the length of hospital stay during the acute COVID-19 episode and the FEV1 value measured at the post-COVID-19 visit. Patients with a longer stay had lower FEV1 values (*p* = 0.013), but no significant relationship was observed with the other measured lung function variables. The Tiffeneau index value differed significantly between the age group of 21 and 30 years and the age group of 51 and 60 years. The DLCO value varied significantly between the age group of 41 and 50 and the age group of 71 and 80. Additionally, the DLCO value differed significantly between the age group of 51 and 60 and the age group of 71 and 80. (Table S1). 

### 3.2. CAT Score

In total, 107 patients filled out the CAT questionnaire. A total of 26 patients (24%) had a low score (0–8) with an average value of 4.7. A medium score (9–24) was marked by 63 patients (59%) with an average of 16.7 points. A high score (25–40) was marked by 18 patients (17%) with an average of 28.7 points. There was no significant correlation between lung function values and CAT scores. 

### 3.3. Post-COVID-19 Symptoms

Detailed data were available for 80 people regarding the symptoms they experienced upon arrival at the PCOS. Out of these, 60 patients (75%) mentioned respiratory symptoms as their leading cause of visit, with general symptoms like fatigue also being frequently reported (73.75%). Pain was reported by 26 patients (32.5%). Besides these symptoms, patients presented with other various organ dysfunction-related symptoms (Table 3).

**Figure 3:**
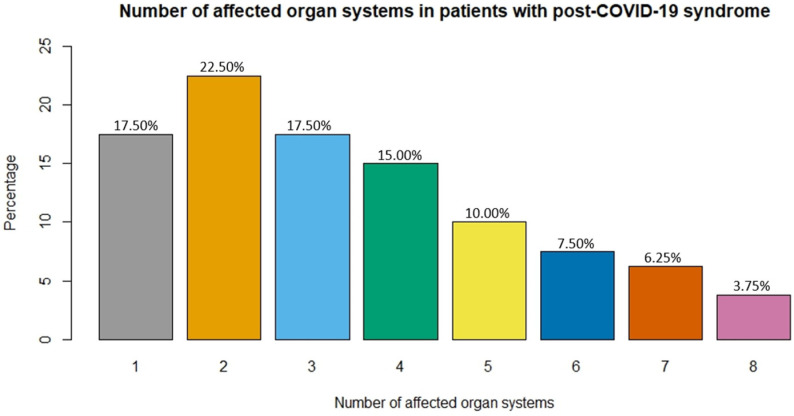
Number of affected organ systems in patients with post-COVID-19 syndrome.

Out of these patients, only 14 (17.50%) of them exhibited symptoms affecting only one organ system, and a large majority of them (82.50%) had dysfunctions of two or more organ systems. A total of 18 patients (22.50%) had symptoms affecting two, 14 (17.50%) had symptoms affecting three, and 12 (15%) had symptoms affecting four organ systems, while 22 patients (27.50%) experienced symptoms affecting more than four organ systems (Figure 3). 

**Figure 4:**
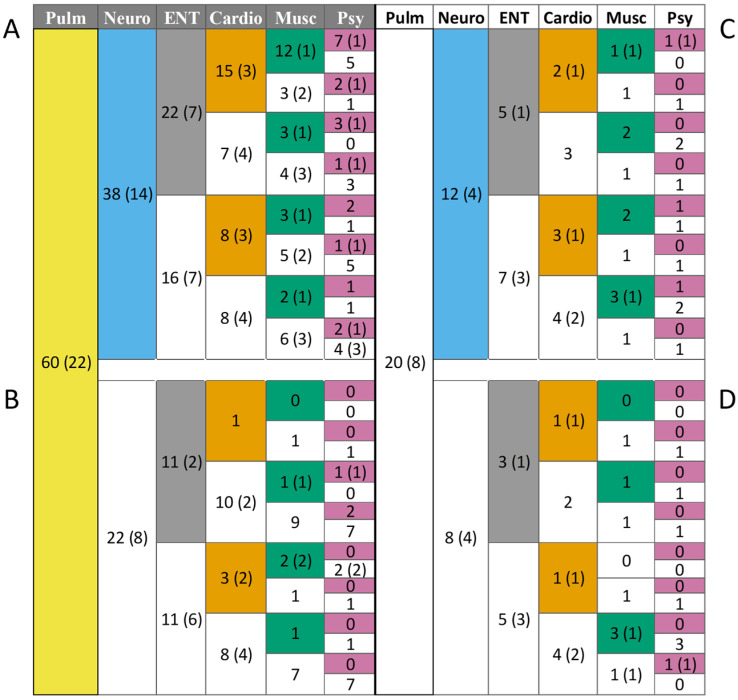
The possible subgroups of post-COVID-19 syndrome. (**A**,**B**) Patients with pulmonary symptoms. (**C**,**D**) Patients without pulmonary symptoms. The first column (yellow) shows patients with pulmonary symptoms, the second column (blue) shows patients with neurological symptoms, the third column (grey) shows patients with ear-nose-throat (ENT) symptoms, the fourth column (orange) shows patients with cardiovascular symptoms, the fifth column (green) shows patients with musculoskeletal symptoms, and the sixth column (purple) shows patients with psychiatric symptoms. Colors indicate the presence of a specific symptom, and white indicates the absence of a specific symptom. Numbers indicate the number of patients, where numbers in parentheses indicate the number of male patients.

We performed a subgroup analysis based on the number of affected organ systems and created four possible distinct subgroups of post-COVID-19 syndrome. At first, we separated patients based on the presence (Figure 4A,B) or absence (Figure 4C,D) of pulmonary symptoms. Further separation was based on the presence of neurological symptoms, the second most frequent in our patients (Figure 4A,C shows the number of patients with neurological symptoms, while Figure 4B,D shows number of patients without neurological symptoms). The appearance of a co-occurrence of symptoms of other organ systems included ear-nose-throat (ENT) symptoms, cardiovascular symptoms, musculoskeletal symptoms, psychiatric symptoms, and gastrointestinal symptoms. Based on these categories, from those who had pulmonary symptoms, 38 patients (63%) also had neurological symptoms; in this subgroup, other co-morbidities were also frequent (Figure 4A): 22 (37%) patients had ENT symptoms, 15 (25%) patients had cardiovascular symptoms, 12 (20%) patients had musculoskeletal, and 7 (12%) of them had psychiatric symptoms. In our post-COVID-19 cohort, eight patients (10%) had seven or more affected organ systems (Table S2).

On the contrary, there were only eight (10%) patients in our cohort, who did not have either respiratory or neurological symptoms (Figure 4D). In this subgroup, only two patients (25% of those not having either respiratory or neurological symptoms) had more than one organ involved in their post-COVID-19 symptoms, and none of them complained about more than two organs (Figure 4D).

### 3.4. Life Quality Assessment

**Figure 5:**
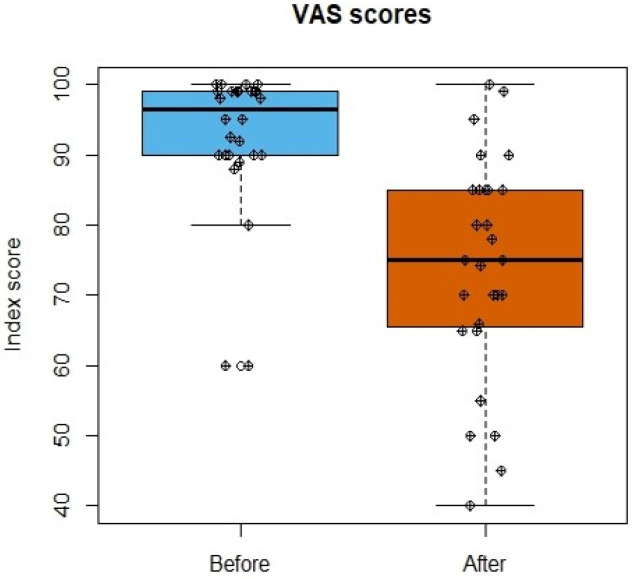
VAS scores before and after SARS-CoV-2 infection. Individual scores are represented by black dots.

To measure the quality of life, we utilized the Visual Analog Scale. The completion of the VAS questionnaire was voluntary; a total of 27 patients filled it out. Patients rated their quality of life at an average of 92.52% before COVID-19 and 74.19% after COVID-19, indicating an average decrease of 18.33%. A significant difference was observed in the quality of life before and after COVID-19 (*p* < 0.001) (Figure 5). 

### 3.5. Smoking Habits

The description of patients’ smoking habits was voluntary during the Pulmonary Clinic visit. We received information on this from 15 patients. Among them, 1 was an active smoker, while 14 had quit. On average, 3.8 ± 2.7 organs were affected in former/active smoker cases, while for never-smokers (and patients without smoking data), 3.4 ± 1.8 organs were affected (see Table S3). 

### 3.6. Vaccination Status

The vaccination data were presented by patients on their vaccination cards, and we were able to track their vaccination records as long as they were under treatment at our clinic.

Before contracting COVID-19, a total of 6 individuals received vaccinations. On average, 2.3 ± 0.8 organs were affected in their cases. After their COVID-19 infection, 30 patients received vaccinations, with an average of 3.5 ± 1.9 affected organs. Only 3 individuals received a booster shot, with an average of 5 ± 2.6 affected organs. Since we could only track vaccinations until patients participated in some examination at the clinic, the rate of booster vaccinations is likely higher. Forty-four individuals had not been vaccinated at all until the end of the study, with an average of 3.6 ± 2.1 affected organs (see Table S3).

### 3.7. Clinical Severity Spectrum of COVID-19 versus Functional Lung Parameters in Post-COVID-19

We classified our patients according to the 2023 COVID-19 treatment guidelines [21] into the following groups: presymptomatic infection, mild, moderate, severe, and critical illness. In the examined patient population, patients were present in every severity category, except for the critical group. The moderate and severe groups were more associated with older age, whereas the presymptomatic and severe groups were particularly characteristic of females. Smoking was strongly associated with the severe group. Lower FEV1 was characteristic of the moderate and severe groups. The Tiffeneau-index did not show a trend among severity groups (see Table S4). 

### 3.8. Co-Morbidities

In total. 22 months after the completion of our investigation, we re-evaluated the data of the 80 patients for whom we had comprehensive mapping of organ involvement. Among the patients, respiratory (26.5%) [asthma (13.75%), COPD (12.5%)], circulatory (37.5%) [ischemic heart disease (5%), heart valve disease (2.5%), hypertension (10%), cardiac arrhythmia (20%)], immunological (12.5%), oncological (5%), metabolic (2.5%), and renal (2.5%) diseases were detected that were not diagnosed (or at least noted on medical records) before the acute episode of COVID-19.

## 4. Discussion

Our results demonstrate that a large majority of patients hospitalized for acute COVID-19 have breathlessness or cough accompanied by fatigue as leading post-COVID-19 symptoms. Based on the pattern of organ dysfunctions, those suffering both from respiratory and neurological symptoms are more likely to have other organ function losses as well. On the other hand, patients having no complaints related to their respiratory and neurological functions are more likely to have only mono-organ dysfunction. Multiple organ involvement is associated with a decreased quality of life.

Our observations are in line with publications from other centers demonstrating that breathlessness, fatigue, and cognitive dysfunctions are the most frequent symptoms in long COVID followed by a wide range of functional losses in other organs [1,2,3,4,7,22,23,24,25].

Due to the fact that our study is retrospective and single-centered with a limited number of patients with a comprehensive assessment of multiorgan involvement, its potential applicability to a wider population is somewhat limited, but it allows us to elaborate on its position between other lines of evidence related to post-COVID-19 and have some clinically practical and important conclusions. 

In the systematic review on the post-COVID-19 topic written by Long and colleagues [26], which included European and Asian sources, the symptoms we observed were consistent. Therefore, we suggest that in general, symptoms are race-independent. At the same time, some lines of evidence point towards different features of acute COVID-19 in Roma patients, and an increased risk of adverse outcomes in migrants and ethnic minorities, compared with the general population [27,28]. The observed differences suggest that the post-acute sequelae of COVID-19 may be somewhat different in these people. In the region surrounding our center, the population is almost exclusively Caucasian with approximately 6–7% of them belonging to a Roma ethnic minority; however, we were not able to assess ethnicity in our work because such sensitive data were not gathered. We urge further studies on this area to enable health care systems to act appropriately and close the health gap mitigating the potentially more severe outcomes in these vulnerable groups.

Our data extend previous observations by providing subgroups based on the pattern of involvement of different organs in post-COVID-19. Our concept points out that a combined persistence/appearance of respiratory and neurological symptoms may represent a warning signal for likely dysfunctions of other organ systems as well. On the contrary, if these two organ systems work normally, multiorgan involvement has a lower likelihood. The exact value of the combined respiratory and neurological involvement to predict multiorgan disease, however, cannot be established in our study because of its cross-sectional nature and relatively small sample size. At the same time, this or other types of subgrouping patients with post-COVID-19 syndrome may open the way to search towards targeted biomarkers and treatment options also suggested by others [29].

Since the symptoms we observed have also been observed elsewhere [26], our classification could be applicable if attempted in populations of different races. Detailed data, however, are not available in that publication to use them as a validation dataset and assess the value of our subgrouping. Our subgrouping system has a very pragmatic route: medical personnel under very heavy time-pressure may identify those expected to have the largest disease burden requiring more health care. For these patients, due to the nature of their complex multimorbidity patterns, it is most important to provide well-organized multidisciplinary care and patient pathway. Since this is the first attempt at creating such subgroups, further investigations may be necessary to assess the comprehensiveness and applicability to the wider patient population to further define the potential utility of our tool.

Furthermore, there is no simple explanation for this combination of organ involvement despite a strong effort of the research community aiming at understanding the post-infective sequelae following acute COVID-19 episodes [30,31,32]. Previous studies demonstrating inflammatory, autoimmune mechanisms, endothelial disease, macrophage activation-related pathways, and virus persistence are all listed as potential causes [30,31,32,33,34,35]. Albeit the concept of having endothelial disease behind is catchy, that cannot explain the principal involvement of the lungs and the brain. The preferential route of virus entry through the respiratory tract together with a more intense presence of angiotensin-converting enzyme (ACE) receptors in specific areas of these two organ systems may represent a possible explanation. This suggestion is supported by our observation that the third most frequently detected dysfunctions occur in the upper airways. The pulmonary dysfunction limiting oxygen uptake and the inflammatory processes activated during the acute episode of COVID-19 causing oxidative stress may have direct consequences on the brain tissue post-COVID-19 and indirect ones through the effect of an imbalance of oxidative and antioxidative activities in the cardiovascular system, as is the case in COPD [32,36]. This together with possible direct damage of neuronal networks by hypoxic episodes could also play a part in the co-existence of neurological damage with respiratory symptoms. Dysfunctional neuronal circuits can further limit respiratory adaptation. 

The observed subgroups of post-COVID-19 organ involvements further highlight the need for more studies on the potential differences in their biomarker profiles aiming at more specific therapeutic options for different subgroups. 

Older age and female gender were previously identified as correlates for a greater risk of persistent COVID-19 symptoms. Our study demonstrates similar results. While high occurrence of post-COVID-19 in elderly can be explained by several lines including the observation that mainly older patients require hospitalization for acute COVID-19, and they have multiple chronic diseases. However, the predominance of females is not that easy to understand. Besides general assumptions related to female hormones, the smaller size of the lungs causing relatively more intense exposure to the viruses could provide plausible explanations; other factors including different communication of symptoms and differences in care-seeking behavior may also play a part.

Based on our data, it seems that both vaccination status and smoking habits might influence the number of affected organs in individuals. However, it is crucial to recognize that these observations are drawn from a relatively small sample size. Various other factors could potentially contribute to the number of affected organs, and a more in-depth analysis would be necessary to determine any significant relationships between vaccination status, smoking habits, and the number of affected organs.

According to our available pulmonary function test results, FEV1 levels were lower after moderate and severe COVID-19 infections, while changes in the Tiffeneau-index did not show a correlation with the severity of previous COVID-19 infections, as a previous study has shown [37]. 

In our study, only a limited number of patients completed the quality-of-life questionnaire (VAS); its results, however, align with findings of others [10]. They point towards the notion that even with less severe dysfunctions in individual organs, patients are more symptomatic if they have multiple organ involvement. Our VAS score was not validated specifically for post-COVID-19; however, in early 2021, there was a great need to use any available methods to serve the pressing need in patient care. It was also the case for the use of the CAT score in these patients that only had its validation while already used in our work. The results of our work are consistent with the CAT questionnaire (and pulmonary function tests) results in another post-COVID-19 study [38]. 

By now there is an increasing knowledge on other potential means of measurement that can be considered, such as the Post-COVID-19 Functional Status scale [39]. 

Although predictive algorithms have already been developed for assessing the risk of acute COVID-19 pneumonia and the likelihood of requiring hospital care [40], there is still no comprehensive method for evaluating the risk of development and implementing complex treatment for long COVID [41]. 

The management of long COVID in primary and secondary care differs from one country to another. Typically, primary care, including GPs and healthcare professionals, is the initial point for assessing long COVID patients, involving a thorough examination of diverse symptoms. However, in certain countries like the UK, USA, and the Netherlands, previously hospitalized COVID-19 patients are directed to secondary care for follow-up consultations conducted through phone or video, concentrating on symptom assessment, and excluding life-threatening or non-COVID-19-related conditions. In our study, it appeared that patients after hospitalization return with post-COVID-19 symptoms relatively late, limiting the potential of interventions. It is not known which individuals will experience full recovery and which individuals will experience a persistence of symptoms after hospitalization. Keeping in mind the long-standing multiorgan dysfunctions in a large number of patients, we support the notion that planned regular follow-ups need to be started early for patients discharged from hospitals after acute COVID-19 [42]. Furthermore, our data draw the attention to the need for a multidisciplinary approach and educational need for care givers to be prepared for monitoring and treating multiorgan dysfunctions. A growing body of evidence shows that the active use of comprehensive, well-targeted rehabilitation is able to enhance recovery from post-COVID-19 [42,43]. Although there is no established curative therapy for it, further studies to characterize its different clinical and biochemical subgroups could facilitate the development of targeted therapies [29,44,45].

Our data show that the average age in our patient population was 55 years of age, with slightly more women included, and predominantly, respiratory and cardiological symptoms were present in the medical history. These results, as risk factors, have already been identified in a systematic review [46], except for age. We did not find any additional risk factors. Further genetic and environmental studies would be needed to address this question more comprehensively.

Several months after the completion of our investigation, we re-evaluated the data of patients for whom we had comprehensive mapping of organ involvement. Among the patients, respiratory, cardiovascular, immunological, oncological, metabolic, and renal diseases were detected that were either newly developed or only newly detected (not diagnosed or at least noted on medical records) before the acute episode of COVID-19. Others also found new-onset neurodegenerative diseases as long-term sequelae of SARS-CoV-2 infection, pointing towards the need of increased awareness of potential COVID-19-driven onset of different disorders [47].

## 5. Conclusions

In summary, post-COVID-19 syndrome is a complex and multifaceted challenge to understand. Subgroup analysis emerges as a powerful tool in unraveling this complexity, offering insights that drive personalized medicine. Subgroup analysis may uncover genetic, environmental, or lifestyle factors contributing to the syndrome. This knowledge not only enhances our understanding of the disease but also guides towards a more effective treatment, ultimately improving the lives of patients worldwide.

## Figures and Tables

**Table 1:** Prevalence of concomitant diseases.

Factors	Values
Age (years; mean ± SD)	55.16 ± 13.1
Male	110 (46) *
Female	128 (54) *
Respiratory diseases	47 (20) *
Asthma	35 (15) *
COPD	11 (5) *
Allergic rhinitis	4 (2) *
Lung cancer	1 (0.4) *
Pulmonary Hypertension	2 (0.8) *
Psychiatric conditions	8 (3) *
Cardiovascular diseases	106 (44) *
Ischemic heart disease	20 (8) *
Heart valve disease	5 (2) *
Hypertension	94 (39) *
Cardiac arrhythmia	10 (4) *
Oncological diseases	14 (6) *
Diabetes mellitus	18 (8) *
Renal disease	1 (0.4) *

* Values are given as absolute numbers with percentage of total in brackets.

**Table 2:** The spirometric results of patients with post-COVID-19 syndrome.

		Number of Patients	% of Patients	Lung Function as % of Predicted Value *
	All	191	100	95.00 (82.00–105.00)
FEV_1_	Low	38	19.90	71.00 (62.25–75.00)
	Normal	153	80.10	98.00 (91–108)
Tiffeneau-index	All	187	100	78.95 (74.16–82.70)
	Low	110	58.82	75.06 (68.97–77.89)
	Normal	77	41.18	84.06 (81.67–88.31)
Dlco	All	59	100	86.00 (73.00–96.50)
	Low	23	38.98	67.00 (61.00–75.00)
	Normal	36	61.02	94.50 (86.75–101.25)

* Data are given as median (IQR).

**Table 3:** Symptoms listed by patients with post-COVID-19 syndrome.

	Number of Patients	%
Total Number of patients	80	100
General pain	26	32.50
Fatigue	59	73.75
Pulmonary symptoms	60	75.00
Shortness of breath, dyspnoe	39	48.75
Cough	37	46.25
Neurological symptoms	50	62.50
Sleep disorder	29	36.25
Cognitive disorder	20	25.00
Peripheral neuropathy	12	15.00
Headache	22	27.50
Dizziness	21	26.25
Delirium	1	1.25
Ear-Nose-Throat symptoms	41	51.25
Earache	8	10.00
Tinnitus	16	20.00
Pharyngalgia	16	20.00
Hearing loss	11	13.75
Loss of smell	7	8.75
Loss of taste	5	6.25
Cardiovascular symptoms	34	42.50
Chest pain	31	38.75
Palpitation	5	6.25
Musculoskeletal symptoms	35	43.75
Myalgia	26	32.50
Arthralgia	25	31.25
Psychiatric symptoms	25	31.25
Anxiety	10	12.50
Nightmares	6	7.50
Depression	7	8.50
Gastrointestinal symptoms	21	26.25
Diarrhea	9	11.25
Abdominal pain	12	15.00
Decreased appetite	6	7.50
Nausea	6	7.50
Dermatological symptoms	6	7.50
Dry skin	1	1.25
Itching	1	1.25
Hair loss	3	3.75
Skin rash	3	3.75
Other symptoms (vision impairment, tremors)	3	3.75

## Data Availability

The data presented in this study are available on request from the corresponding author.

## Supplementary Materials

The following supporting information can be downloaded at: https://www.mdpi.com/article/10.3390/jcm13051457/s1, Table S1: *p*-values of different correlations; Table S2: Combination of symptoms in patient cohort; Table S3: Smoking habits and vaccination status of patients; Table S4: Clinical severity spectrum versus functional lung parameters.
